# Inorganic and Hybrid (Organic–Inorganic) Lamellar Materials for Heavy Metals and Radionuclides Capture in Energy Wastes Management—A Review

**DOI:** 10.3390/ma12091399

**Published:** 2019-04-29

**Authors:** Marie Claverie, Justo Garcia, Thierry Prevost, Jocelyne Brendlé, Lionel Limousy

**Affiliations:** 1 Institut de Science des Matériaux de Mulhouse CNRS UMR 7361, Université de Haute-Alsace, Université de Strasbourg, 3b rue Alfred Werner, 68093 Mulhouse CEDEX, France; jocelyne.brendle@uha.fr (J.B.); lionel.limousy@uha.fr (L.L.); 2 Orano, Tour Areva, 1 place Jean Millier, 92400 Courbevoie, France; justo.garcia@orano.group (J.G.); thierry.prevost@orano.group (T.P.)

**Keywords:** selective adsorption, radionuclides, heavy metal, phyllosilicates, LDH, decontamination, clean processes

## Abstract

The energy industry (nuclear, battery, mining industries, etc.) produces a large quantity of hazardous effluents that may contain radionuclides (^137^Cs and ^90^Sr in particular) and heavy metals. One of the hardest tasks of environmental safety and sustainable development is the purification of wastewater holding these pollutants. Adsorption is one of the most powerful methods for extracting toxic compounds from wastewater. This study reviews the usefulness of clay minerals as adsorbent for removing these hazardous elements to clean up energy production processes. Phyllosilicates are able to extract several heavy metals from effluent, as widely examined. A particular focus is given to synthetic phyllosilicates and their abilities to entrap heavy metals with a special attention paid to those synthesized by sol-gel route. Indeed, this method is attractive since it allows the development of organic–inorganic hybrids from organosilanes presenting various functions (amino, thiol, etc.) that can interact with pollutants. Regarding these pollutants, a part of this review focuses on the interaction of lamellar materials (natural and synthetic phyllosilicates as well as layered double hydroxide) with heavy metals and another part deals with the adsorption of specific radionuclides, cesium and strontium.

## 1. Introduction

The issue of radionuclides and heavy metals contamination, their consequences on population health and the ecosystem have been the major health concerns in recent years as they are a major source of ill health, producing short and long-term side effects and sometimes irreversible brain damage, kidneys and other organs despite treatment [1,2,3]. Water pollution is one of the colossal environmental subjects, as it can induce significant damages to living beings. Over the past decades, several scientists and researchers have paid particular attention to removing various toxic substances from water and wastewater. Bountiful energy industries, such as nuclear power operations, battery, oil and mining industries, may produce contaminated wastewater effluent containing myriad kinds of toxic wastes such as heavy metals and radionuclides [2,4]. Accordingly, the unique remedy is to eliminate them from the contaminated effluent before they are released into the ecosystem.

Numerous methods, using biological, physical or chemical processes, have been proposed for wastewater treatment. Biological methods generally require large spaces and are not applicable to all pollutants. Chemical processes, such as evaporation, ion exchange, chemical precipitation, membrane filtration technologies, etc. [5,6,7,8,9,10,11,12], are generally very expensive and not suitable for low pollutant concentrations and selective removal, which causes damages to both the environment and life [13].

Adsorption is the physical method considered as the cheapest and most effective solution [14,15,16,17,18]. The technique is based on the split of an element from one system (liquid phase for example) and its concentration in another system (at the surface of a material for example). This approach, mainly used for volatile organic compounds [19], heavy metals and ammonia treatment, is easy to achieve and effective in eliminating poisonous pollutants, at low concentration [20]. The adsorbent choice is one of the most decisive steps in any adsorption mechanism. Activated carbon and clays are the two most commonly used adsorbents in solution adsorption [21]. However, activated carbon has higher production and regeneration costs than clays.

For decades, clays and clay minerals have been attractive for extracting various noxious heavy metals and radionuclides from water considering their versatile properties, their structural and chemical composition diversities, their biocompatibility, their environmental safety, their natural abundance and finally their low-cost [3,22].

Several studies were conducted to determine the adsorption capacities on radionuclides, heavy metal cations or certain persistent organic contaminants using various kinds of natural, modified, functionalized or synthetic phyllosilicates [23]. Most phyllosilicates are greatly preferred to adsorb metal elements from solutions thanks to the occupation of interchangeable cations in the space between the layers, their great pore volume and surface area [24,25,26,27]. The uptake of toxic substances by phyllosilicates covers a succession of intricate adsorption processes, e.g., straight contact between the phyllosilicate surface and metal cations, ion exchange and surface complexation [28]. In several papers, phyllosilicates are pre-treated to boost adsorption power [29]. These treatments render clays hydrophobic, i.e., organophilic, which improves the absorption of small non-ionic organic elements [30].

This review presents an outline of the adsorption behavior of natural and organically modified clays and a focus on the synthesis of innovative organic–inorganic hybrid phyllosilicates obtained by sol-gel method, which may be good candidates for the radionuclides and toxic pollutants adsorption. The first part is dedicated to the heavy metals adsorption by natural phyllosilicates (modified or not), by synthetic organic–inorganic hybrids having a phyllosilicate-like structures and by layered double hydroxides (LDHs). The second part is devoted to radionuclides adsorption (mainly cesium and strontium) by phyllosilicates and LDHs.

## 2. Use of Lamellar Materials for the Purification of Aqueous Solutions Containing Heavy Metals

### 2.1. Natural Clay or Modified Natural Clay and Heavy Metals Adsorption

Several studies have explored the adsorptive profiles of natural clay minerals and chemically modified clays (more than 300 publications listed). In these studies, adsorption processes are highly dependent on solution pH, element concentration, proximity time, etc.

#### 2.1.1. Classification, Structure and Properties of Clays

**Figure 1:**
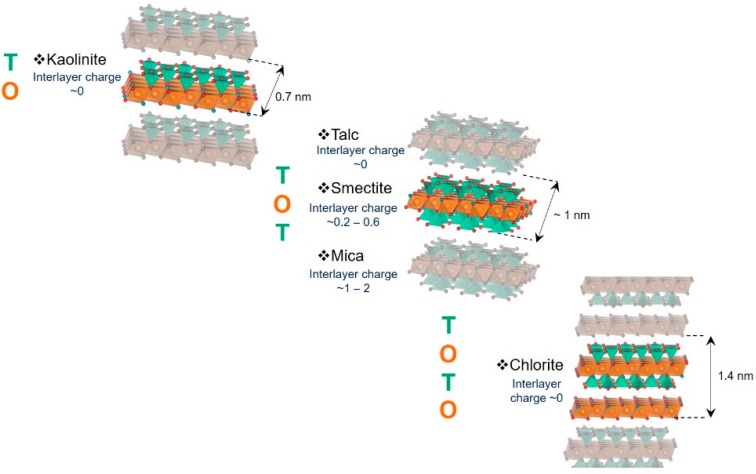
Representation of the three main phyllosilicate families.

In nature, clay minerals come from the alteration of rocks. Generally, the term “clay” refers to sedimentary rocks composed mainly of clay minerals, differentiable through several structural and chemical parameters. Technological advances in solid analysis have enabled these clay minerals to be characterized and classified according to their layer thickness into four main families (Figure 1) [31].

- **Minerals of type 1:1**. They are also called 7 Å minerals because of the thickness of the layer (composed of an arrangement of tetrahedral silicate sheets with octahedral hydroxide sheets). They consist of a siliceous tetrahedral (T) sheet bounded to an aluminous octahedral (O) sheet, or a T.O. stack, another name given to this family of clay minerals. Kaolinite is a notable mineral of this group. In the case of halloysites, the interlayer space can be occupied by water molecules.
- **Minerals of type 2:1**. This family of minerals (also named T.O.T. type) consists of an arrangement of one octahedral sheet sandwiched between two silicate sheets and presents a layer thickness close to 10 Å. It is the most common type of phyllosilicate encountered in nature. This family includes mica, smectite and illite, which could be distinguished by the contents of their tetrahedral and octahedral sheets. Some members of this family have swelling properties. While smectites and vermiculites can swell by incorporating water molecules, or hydrated cations, into the interlayer space, most micas or illites having interlayer potassium cations have no ability to swell.
- **Minerals of type 2:1:1**. They have a T.O.T.O. structure with a layer thickness of about 14 Å. For this family of clay minerals, the interlayer space is occupied by an octahedral sheet. Chlorites are part of this family of minerals.
- **Interlayered minerals**. This last family is characterized by its heterogeneity. Indeed, interlayer minerals have diverse kinds of layers that could be stacked regularly or not. In nature, interlayered minerals illite/smectite or mica/chlorite are frequently found.

The dissolution kinetics of clays in solution are controlled by different parameters: temperature, particle size, presence of impurities and pH.

**Figure 2:**
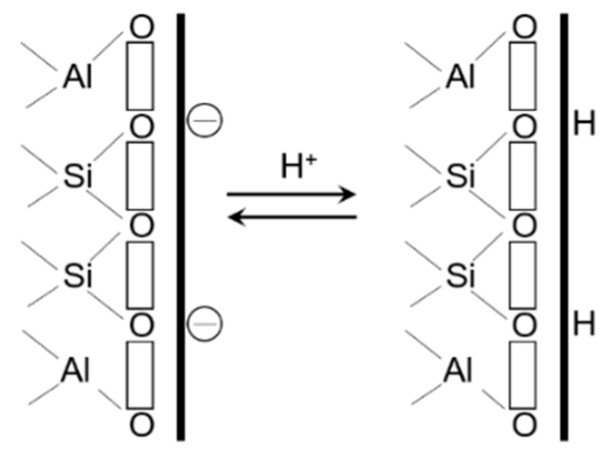
Reaction on the basal surface of a clay mineral. Reprinted with permission from reference [33].

**Figure 3:**
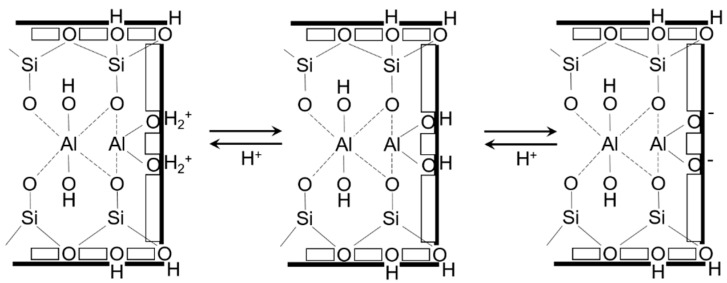
Reaction at the edge of phyllosilicate layers. Reprinted with permission from reference [33].

While usually very slow, this kinetics can be accelerated by the existence of hydrogen ions in the phyllosilicate by causing aluminum and silicon removal toward the solution. Nevertheless, the slowness of these phenomena allows considering clays as metastable materials. Concerning the surface reactivity of clay minerals, it involves different types of interaction [32]:

- Negative charges relocated at the layer surfaces (basal surfaces) make these surfaces good sites for cation retention by charge compensation (they result from isomorphic substitutions which then lead to deficits of positive charges) (Figure 2).
- Layer edges are composed of silanol and aluminol groups, which, depending on pH, can deprotonate (or even protonate). These changes result in the presence of positive (or negative) charges which can further be neutralized by complexation (Figure 3).
- Exchangeable cations can be supplanted by diverse cationic elements, the interaction between these latter and the surface being electrostatic. Interlayer spaces containing exchangeable cations are negatively charged surfaces that can interact with different cations in a very specific way (electrostatic interactions).

Cation exchange capacity (CEC) is a key to identify phyllosilicates reactivity. It quantifies the adsorption ability of a mineral. Determined by measuring the adsorption of charge-compensating cations (such as ammonium NH_4_^+^, cobaltihexamine or calcium Ca^2+^ ions), CEC is generally expressed as the amount of charge or number of moles adsorbed per unit mass of adsorbent (mmol·g^−1^). Given the above-mentioned types of reactivity, in particular because of protonation/deprotonation mechanisms of silanol and aluminol groups, the CEC value is linked to the solution pH. Thus, the higher is the pH, the greater is the adsorption of cations, and the higher is the CEC value. The maximum CEC is thus obtained at alkaline pH.

Phyllosilicates are minerals encountered in all environments on the globe surface (continental or marine). Their small size gives them high-specific areas and therefore great reactive properties towards the environment (water, air, etc.) in which they are in contact. Among these properties, those concerning adsorption/desorption of solute make them very interesting in the field of purification. The purpose of the following section is to review the studies conducted on the adsorption of heavy metals by phyllosilicates.

#### 2.1.2. Summary of Uddin’s Review on the Retention of Heavy Metals by Natural Clay Minerals

Due to the space between their layers, phyllosilicates attract toxic substances from wastewater. Smectites can swell and increase their interlayer distance to adjust water containing ionic elements.

Uddin recently published an exhaustive review that collects extensive literature of ongoing research over the past ten years (2006–2016) and underlines the major discoveries of adsorption research using phyllosilicates as adsorbent [3]. This review paper introduces an overview of their adsorption performance. It attests that both natural and adjusted structures of phyllosilicates have admirable usefulness for the extraction of various heavy metals. In most cases, adsorption analyses are performed using batch method to indicate the best adsorption efficiencies against focused noxious elements (adsorption isotherms), approving their relevance and selectiveness. The performance of phyllosilicates and the adsorption mechanism have been confirmed by exploring the consequence of many factors such as pH, temperature, concentration, dosage, etc.

For comparison purposes and to define the comparative reviews on the adsorption efficiency of different phyllosilicates, Table 1 highlights the best adsorption capabilities of different phyllosilicates. Thus, the author claims that treated bentonite is more efficient for cadmium adsorption than the others. Modified Montmorillonite reveals higher adsorption for mercury and chromium than the others. Kaolin is preferred for zinc, nickel, or lead adsorptions. Studies on goethite have shown excellent adsorption of arsenic. Subsequently, it is conceivable to accept that the untreated Smectite group (montmorillonite, smectite and bentonite) is the optimum class to take up many metal elements. However, this review does not mention research topics on the heavy metals adsorption on synthetic phyllosilicates. As a result, the following section completes the work done by Uddin.

### 2.2. Heavy Metals Removal by Synthetic Phyllosilicate

One of the future research trends of innovative components derived from nanotechnology for ecological facets is highlighted by the synthesis of organic–inorganic hybrids having a phyllosilicate-like structures. The synthesis pathway brings new advantages such as a high purity level and a possibility to chemically modify it at any stage of the synthesis [33,34]. The literature lists one major synthesis pathway related to heavy metal adsorption: the sol-gel method.

**Figure 4:**
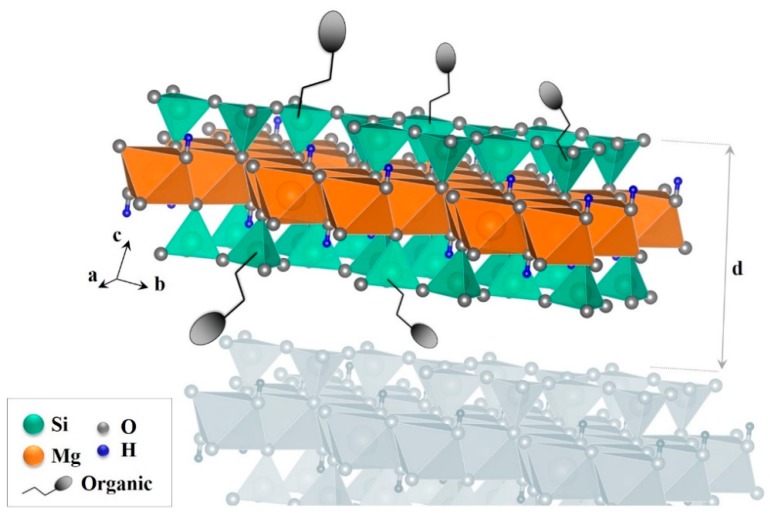
Structure of a standard organic–inorganic phyllosilicate obtained with a sol-gel process: d is the layer plus interlayer thickness.

The sol-gel process is reported as a “soft chemistry” due to ambient reaction temperature. Indeed, low temperature is prescribed with regard to the organic nature of the precursors. Inorganic–organic hybrid phyllosilicates are obtained through the polymerization of hydrated salts (e.g., magnesium chloride) and alkoxysilane (e.g., tetraethoxyorthosilicate, TEOS) or an organoalkoxysilane corresponding to chemical formula RSi(OR’)_3_ (R being an organic group and R’ an ethyl or a methyl moiety) under alkaline conditions and at room temperature or at temperature below 100 °C [43,44,45,46,47,48]. Several factors influence the sol-gel synthesis such as pH, nature of solvent, catalyst and metallic precursor, temperature and concentration [49,50,51]. In 1995, Fukushima and Tani were pioneers in sol-gel synthesis of organic–inorganic hybrids exposing a talc-like network represented by (RSi)_4_Mg_3_O_8_(OH)_2_ (Figure 4) [52,53]. Unlike the grafting of organic compounds on phyllosilicates via -OH sites located on phyllosilicates edges (and requiring the use of toxic solvents such as toluene), the synthesis of organo-phyllosilicate makes it possible to increase the number of organic moieties since they are fixed on the particle edges and surfaces.

**Figure 5:**
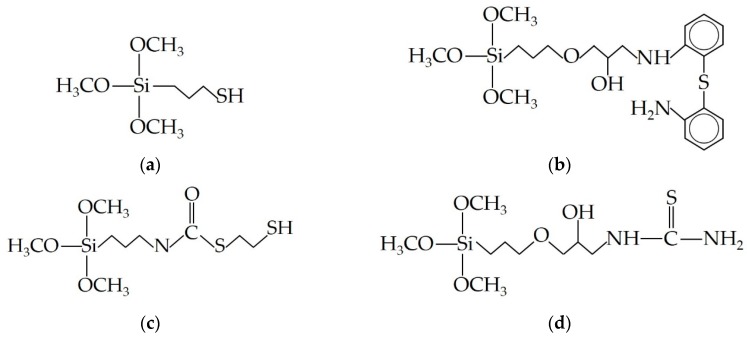
Silylating agents used by: (**a**) Fonseca et al. (3-mercaptopropyltrimethoxisilane); (**b**) Moscofian et al. (silane containing a 2-aminophenyldisulfide molecule); (**c**) Badshah et al. (thiocarbamate organosilane); and (**d**) Dey et al. (organosilane derived from 3-glycidoxypropryltrimethoxysilane and thiourea) in their sol-gel synthesis of phyllosilicates.

Fonseca et al. (2000) used the sol-gel method to form magnesium phyllosilicate (SILMgSH) with a terminal thiol group in the organic moiety (Figure 5a) attached to the inorganic layer (based on talc structure) [46,54]. The objective of this research was to highlight the interaction between this thiol group and divalent cations (nickel, copper, zinc and cobalt in solution). Indeed, due to a soft interaction between a Lewis acid and a Lewis base, thiol functionalization is forecasted to display a connection capacity with heavy metals such as lead, mercury and cadmium [55]. The authors emphasized the formation of more organized compounds with copper and underlined that all other reactions are entropically favored. In the same way, other studies about the sol-gel synthesis of phyllosilicates with organic moieties containing nitrogen linked to the pending organic chains highlight their ability to remove nickel, copper or zinc from aqueous solutions [56,57].

Lagadic et al. (2001) also prepared a 2:1 magnesium phyllosilicate (with a talc-like structure) functionalized with thiol group by using mercaptopropyltrimethoxysilane (named Mg-MTMS) [58]. The material is highly efficient in adsorbing Hg^2+^, Cd^2+^ and Pb^2+^ ions with metal ion adsorption capacities of 603, 210 and 365 mg of metal/g of adsorbent, respectively. Moreover, the metal-loaded Mg-MTMS can be reestablished by acid treatment without modifying the adsorbent abilities. According the authors, both a great quantity of thiol groups (6.4 mmol of SH per gram of Mg-MTMS) and a distension potency of the structure facilitate sites accessibility and are the main reason of this huge performance.

However, based on thiol-functionalization, Jaber et al. (2005) were interested in the sol-gel synthesis at room temperature of a compound highlighting a saponite-like network with the general formula Na_x_[(RSi)_(4−x)_Al_x_Mg_3_O_(8+x)_(OH)_2_ with x ranging from 0.1 to 1.2 [59]. The reaction was performed starting from mercaptopropyltrimethoxysilane, aluminum acetylacetonate and magnesium nitrate. These hybrids show a great adsorption capacity for copper and mercury (~100%). According to the authors, the penetration of pending chains in a neighboring layer thanks to hexagonal cavities located on tetrahedral sheets allow. Nevertheless, cationic migration in octahedral sheet due to mercaptopropyl function induces structure collapse.

Moscofian et al. (2008) chose another alkoxysilane to synthesize a hybrid organotalc through a sol-gel process to adsorb copper, lead and cadmium. Using a 2-aminophenyldisulfide compound reacting with 2-aminophenyldisulfide (Figure 5b) and magnesium nitrate, they obtained a talc with basic sulfur and nitrogen centers inside the lamellar framework (with a great level of functionalization of about 1.97 mmol·g^−1^) allowing to complex these cations from aqueous solution. Copper, cadmium and lead had a maximum adsorption capacity of 3.28, 0.35 and 1.42 mmol·g^−1^, respectively. This hybrid nanomaterial has a cation adsorption capacity comparable to other adsorbent which suggests new promising adsorbent for cations removal from polluted systems. Later, Badshah et al. (2011) presented another organotalc (with basic sulfur and nitrogen centers, Figure 5c) highlighting higher retention abilities for copper, cadmium and lead (4.01, 1.86 and 7.08 mmol·g^−1^, respectively) [60].

A talc-like hybrid synthesized with a new silicon source derived from 3-glycidoxypropyltrimethoxysilane and thiourea (Figure 5d) was proposed by Dey et al. (2009) [57]. This hybrid presents a high lamellar distance (~18.8 Å) due to the existence of organic chains. Metal adsorption of this talc-like has been explored and follows Cr(III) > Mn(II) > Zn(II) with higher attraction towards Cr(III) in dilute watery solution. The authors also highlighted the endothermic character of the Cr(III) adsorption process and its practicability and spontaneousness of continuing operations at relatively higher temperatures. The existence of several ligating sites in this hybrid broadens the area of utilization in metal coordination chemistry.

Other studies are focused on heavy metals adsorption by organotalc obtained through soft sol-gel approach. All these studies (summarized in Table 2) contribute to the development of new research areas on environmental aspects that is presumably to be considered more greatly.

Natural, modified and synthetic clays are materials in full development, particularly interesting because of the improvement of their physicochemical properties, and their interest in the field of adsorption. Indeed, the adsorption capacity of natural clay can be enhanced by modifying its surface using different modifying agents. The synthesis provides control over the composition of the phyllosilicate but also over its properties which can be modulated (such as particle size, structure, etc.). Therefore, the modification and synthesis of phyllosilicate would be beneficial for the preparation of new adsorbents intended to the treatment of water containing pollutants. While the potential of lamellar materials has been demonstrated, there are still some conflicting elements in the literature regarding their respective adsorption capacity and the adsorption reversibility. The following section deals with another lamellar material, also called “anionic clay” due to similar structure to clays: Layered Double Hydroxide (LDH). These porous compounds exhibit attractive properties such as high surface area, which is of interest for water purification. The following section aims to analyze research on the adsorption of heavy metals by LDHs.

### 2.3. Heavy Metals Retention by Layered Double Hydroxides

To complete the research on metallic-trace-element adsorption by lamellar materials, it is interesting to focus on the usage of layered double hydroxides (LDHs) for heavy metals adsorption.

These latter are a group of basic inorganic lamellar materials with an anion in the interlayer space, conducing to interesting anion exchange capacity [65]. The LDHs are also commonly recognized as hydrotalcite-like species due to their structural correlations with the hydrotalcite mineral presenting a chemical formula of Mg_6_Al_2_(OH)_16_CO_3_·4H_2_O [65]. Over the most recent 20 years, there have been plentiful studies on formulation and utilizations of LDHs [65,66,67,68,69,70,71,72,73]. There is presently a growing enthusiasm in LDHs regarding their abilities as catching compounds for anionic contaminants [74,75,76], catalysts [66,72,77], flame retardants [66], polymer stabilizers [66,78], ion exchangers, adsorbents, etc. [79] The first application of LDH for the heavy metals removing was published in 1992 by Fujii et al. through the Mg-Al LDH synthesis including diverse anions in the interlayer used for the adsorption of Zn^2+^, Cu^2+^ and Pb^2+^ [80].

The potentiality of LDH as captors for water decontamination was recently discussed by Zubair et al. [81]. Such as for phyllosilicates, the LDHs application in water decontamination is promising due to their non-toxicity [82], huge surface area, hugely adjustable structure [83], exchangeable anionic characteristics [84] and low-cost. This review introduces an overview of their adsorption performance with a spotlight on the impact of several influential adsorption factors (contact time, temperature, pH, etc.). The greatest adsorption capacities of different heavy metals are reported in Table 3.

The state of the art reveals that the coupling of LDH with different anion (such as polysulfide, and MoS_4_^2−^), polymers, etc. is the main technique used to adsorb heavy metals. These LDHs displays interesting characteristics such as stability and higher selectivity for heavy metals [81,85,86].

The adsorption mechanism involved with these LDH are physical adsorption, anion-metal complexes, chemical bonding, hydroxide precipitation and electrostatic interactions [81]. The heavy metal adsorption of anionic/LDH is mainly governed by hydroxide precipitation and anion-metal complexes. Within the instance of LDH containing carbon based compounds, the physical adsorption and the exchange of interlayer anions (CO_3_^2−^, Cl^−^ and NO_3_^−^) by metal ions (through chemical connection with hydroxyls situated on the carbon compounds surface) are the main adsorption mechanisms.

In their recent study, Li et al. (2018) proposed an innovative multipollutant adsorbent by synthesizing a LDH hybridized with fulvic acid [87]. This compound highlights a surface where fulvic acid (with many functional groups) is bound, and a free interlayer space allowing anionic exchange. This compound therefore allows the adsorption of heavy metals such as copper, nickel, lead and cadmium (at contents of 2.25, 0.99, 0.98 and 0.16 mmol·g^−1^) but also an anionic exchange with Orange II or CrO_4_^2−^. This multipollutant adsorbent is a precursor in the development of materials presenting diverse adsorption sites for diverse kinds of noxious elements.

By comparing these results with previous ones on natural and synthetic phyllosilicates, LDH is an interesting adsorbent for mercury (4.05 mmol·g^−1^). However, chromium, cadmium and nickel are strongly adsorbed by natural phyllosilicates (5.94, 8.64 and 2.40 mmol·g^−1^, respectively). Finally, synthetic phyllosilicates seem to be excellent adsorbents for cobalt, copper, zinc and lead due to their very high adsorption capacities (3.03, 8.09, 6.49 and 7.08 mmol·g^−1^, respectively).

Phyllosilicates and LDH are part of an emerging and pioneering area of research in effluent decontamination. They acquired a renewed interest in water treatment due their high surface area, nontoxicity and stability.

Some energy industries, such as the nuclear-power industry, nuclear medicine, rare earth extraction industry or the Defense sector (construction of atomic weapons), generate various kinds of radioactive effluents for which appropriate treatments are necessary to extract the radioactive elements that contaminate them: mainly strontium 90 and cesium 137. The following section provides a way to extract these two radionuclides through adsorption on phyllosilicate or LDH.

## 3. Use of Lamellar Materials for the Treatment of Effluents Containing Radionuclides

### 3.1. Clay Minerals Radionuclide Adsorption Investigations

The effluents produced by nuclear industries contain mainly two radionuclides, ^137^Cs and ^90^Sr, which have a very short half-life (around 30 years) making them particularly radiotoxic [91,92]. Their selective extraction is therefore a prerequisite for the remediation of these contaminated waters, but it is made difficult by the presence of other alkaline and alkaline earth ions naturally present in these waters. Co-precipitation is used to selectively extract ^90^Sr and ion exchange is used to extract ^137^Cs. Despite good decontamination performance, these processes have the major disadvantage of producing large quantities of sludge, which is the ultimate waste product of these processes. Mineral adsorbents were considered to decrease the volume of final waste.

**Figure 6:**
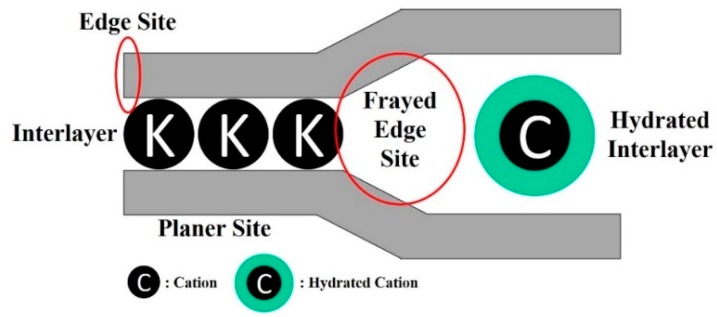
Illustrative representation of the Cs^+^ adsorption positions on 2:1 phyllosilicates. Reprinted with permission from reference [100].

**Figure 7:**
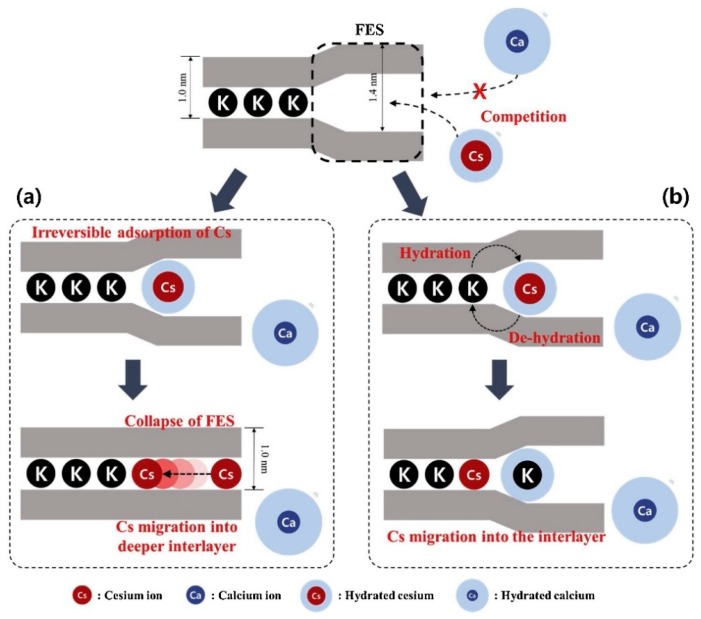
Schematic illustration of the Cs^+^ adsorption sites onto the illite interlayers thanks to: (**a**) the collapse of FES; and (**b**) the dehydration of Cs^+^. Reprinted with permission from reference [100].

In the case of cesium, there are currently materials well known for its extraction (more or less selective) from effluents such as natural or synthetic zeolites, silicotitanates and ferrocyanides [93]. However, scientists have focused on economically viable and environmentally friendly materials: 2:1 type phyllosilicate. Among these clay minerals, montmorillonite (MMT) has a better Cs^+^ adsorption capacity (~780 mmol·kg^−1^) than vermiculite (~270 mmol·kg^−1^) or illite (~150 mmol·kg^−1^) due to its larger surface area, more stable chemical properties, natural pores and high CEC [94,95,96,97,98,99]. In their recent review, Park et al. (2019) studied the selective and non-reversible adsorption of cesium on these type 2:1 clays [100]. Clay minerals present five different sites of adsorption, as illustrated in Figure 6. The authors explained that ^137^Cs “can be reversibly adsorbed on the planar site and on the edges sites or irreversibly adsorbed onto the frayed edge site (defined as the wedge-shaped intermediate zone between non-expansible (10 Å) and hydrated (14 Å) interlayers) and the interlayer” [100,101,102]. Cs^+^ adsorption, migration and irreversible fixation mechanisms on illite are proposed by the authors with two scenarios (Figure 7). The first mechanism proposed is the disorganization of the frayed edge site (FES) by cesium (Figure 7a). Cesium is firstly fixed onto the FES leading to the disorganization of the FES which becomes a normal interlayer site. In this case, the migration of Cs^+^ into deeper interlayer is possible. The second mechanism proposed (Figure 7b) is related to a disparity in moisturization energy of K^+^ and Cs^+^. Due to a K^+^ hydration energy higher than Cs^+^, the Cs^+^ adsorbed into the FES is desiccated and allows the hydration of K^+^ located near the FES. This hydration–dehydration phenomenon allows a change of position between dehydrated Cs^+^ and hydrated K^+^. Cs^+^ is therefore irreversibly fixed in the layer.

Regarding the strontium adsorption, there are few selective strontium adsorbents (mainly against calcium). In this respect, some studies have been developed on inorganic phyllosilicate structure-based cation exchangers for the removal of Sr^2+^.

**Figure 8:**
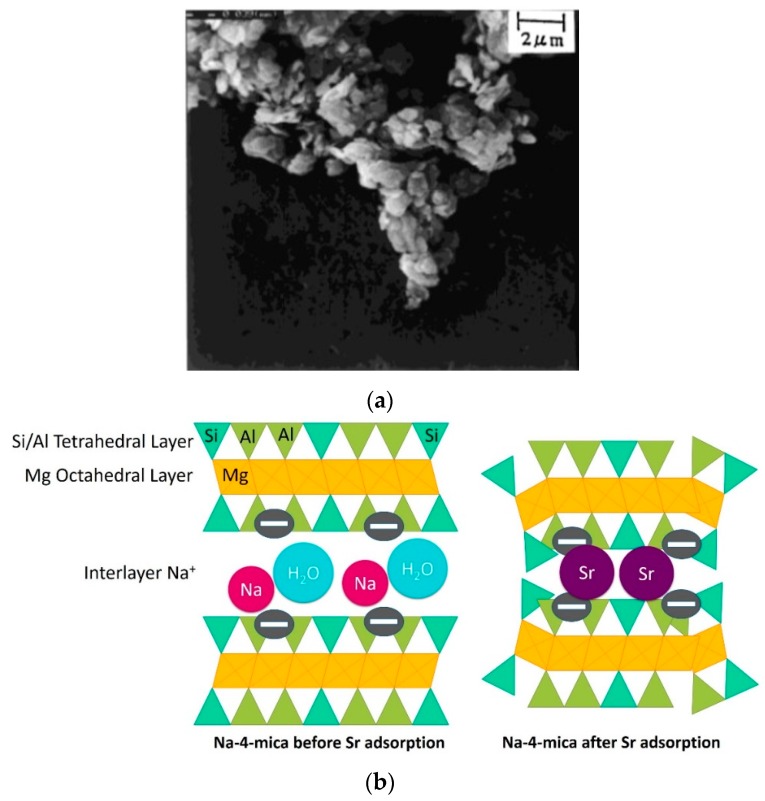
(**a**) SEM micrograph of Na-4-mica synthesized by Kodama et al. [104]; and (**b**) schematic illustration of Sr^2+^ adsorption and immobilization by Na-4-mica. Reprinted with permission from reference [104].

Paulus et al. (1992) took an interest in a synthetic mica (Na-4-mica) to selectively remove strontium ions from effluent [103]. In light of the fine and uniform particle dimension (Figure 8a), the number of available exchange sites is maximized and the interlayer dimensions and charge density are ideal for strontium diffusion and eventual capture. An effective strontium fixation is achieved due to interlayer space collapse. Strontium is captured into a trioctahedral mica network, which is a highly steady phase because of great columbic strengths covering the space, maintaining it tightly closed (Figure 8b). The authors also highlighted that other common divalent ions such as Ca^2+^ and Mg^2+^ (with hydrated radii larger than Sr^2+^) were not trapped between layers. Kodama et al. (2001) were also interested in the adsorption and interchange of Sr^2+^ in Na-4-mica (synthesized with a simpler method) [104]. Following a four-week balancing period, the ion exchange (of two Na^+^ cations by one Sr^2+^ cation) was ascertained and the strontium exchange capacity attained about 2 m_equiv_·g^−1^. It was assumed that Sr^2+^ can be shut in the ditrigonal holes of the Na-4-mica due to high Coulombic interactions. It was suggested that the weak quantity of Sr^2+^ unleashed might be because of those localized on the borders of the interlayers. After the equilibration of four weeks, the non-reversible Na-Sr exchange isotherm was attained at room temperature. According to the authors, this synthetic material is more selective for Sr^2+^ and Ba^2+^ than for Ca^2+^ and Mg^2+^. This particular Sr-ion exchanger should be appropriate for ^90^Sr extraction and its confinement due to a huge ion-exchange capacity and a weak strontium leachability.

The sorption of strontium by natural clays has been studied by some researchers (summarized in Table 4). In 1995, Khan et al. enlightened an endothermic and spontaneous strontium adsorption on a bentonite (from Pakistan) at 298 K due to positive amount of the adsorption heat (ΔH° = 30.62 kJ/mol) and negative amount of the adsorption free energy (ΔG° = −10.69 kJ/mol). They underlined that sorption process is more suitable at warmer temperatures and that ion transfer is the main adsorption mode. Furthermore, the presence of complementary cations reduces strontium adsorption on bentonite according this order: Ca^2+^ > Mg^2+^ > K^+^ > Na^+^. Desorption surveys with groundwater at small Sr loads on bentonite reveal that around 90% of the strontium is irremediably adsorbed onto bentonite.

Missana et al. (2008) took an interest in adsorption behavior of strontium in smectite/illite mixtures (previously homoionized in sodium) by proposing a mechanistic approach (while other studies are mainly based on an empirical approach) [105]. As a result, several parameters were considered such as pH variation, radionuclide concentration and ionic strengths. In this approach, the pH dependence is provided by the sorption on border sites M-OH (silanol or aluminol sites). Adsorption on cation transfer sites does not depend on pH. By contrast, electrolyte cations adsorption exclusively occurred on cation transfer sites. Model predictions are in accordance with experimental adsorption data highlighting a major strontium adsorption contribution of ionic exchange.

^90^Sr(II) adsorption on Na-montmorillonite (Na-MMT) was explored by Yu et al. (2015) under several exploratory parameters (temperature, ionic strength, pH and humic acid) [106]. The authors underlined that this adsorption is hugely reliant on ionic force and pH. In this way, at low pH, “strontium adsorption is dominated by outer-sphere surface complexation and ion exchange with Na^+^/H^+^ on Na-MMT surfaces (and humic acid enhances strontium sorption at pH < 7). In contrast, “at high pH, inner-sphere surface complexation is the main adsorption mechanism” (and humic acid decrease strontium sorption at pH > 7). At last, strontium adsorption on Na-MMT is endothermic and spontaneous.

Siroux et al. (2017) proposed an exhaustive investigation to ascertain Sr^2+^ adsorption on smectite (a pure Na-MX80 montmorillonite) by proposing a multi-site ion exchange modeling [33,107]. This investigation presents a large database which is a powerful tool to predict strontium adsorption in sediments and soils. The authors revealed that strontium adsorption equilibrium is achieved within the first few minutes of contact and that adsorption increases as the ionic strength decreases.

In their paper, Wu et al. (2012) modified a Ca-montmorillonite (Ca-Mt) with 3-aminopropyl triethoxysilane (APTES) or two alkyl surfactants: sodium dodecyl sulfate (SDS) and hexadecyltrimethylammonium bromide (HDTMAB) [108]. The adsorption behavior of Sr^2+^ by organo-montmorillonites was studied in batch experiments. The tests uncovered that the adsorption capability of APTES-Mt is 65.6 mg/g, higher than those of SDS-Mt, HDTMAB-Mt and Ca-Mt (26.85 3.91 and 13.23 mg/g, respectively) under the selected conditions. According to the authors, Sr^2+^ removal by Ca-Mt is predominantly due to ion exchange. Nonetheless, those of APTES-Mt and SDS-Mt are due to ligand sorption and surface sorption, respectively.

To conclude, one of the foremost critical environmental issues in nuclear industry is radioactive wastewater treatment notably containing hazardous radionuclides such as strontium and cesium. The most appropriate solution to recover radionuclides is liquid waste sorption treatment. Some studies deal with strontium sorption on clay minerals (essentially natural), highlighting good adsorption capacities. Overall, different clay minerals exhibit different adsorption behaviors depending on the major cation present. The main interaction in natural phyllosilicates seems to be ion exchange mechanism between strontium and phyllosilicate, while it is ligand adsorption in the case of organically grafted phyllosilicates. However, it would be legitimate to study the long-term resistance of these phyllosilicates. Temperature, pressure and radioactivity of elements can degrade the phyllosilicate structure and cause the release of noxious elements.

Another pathway, similar to Paulus’ study idea on strontium entrapment in interlayer space: radionuclide capture into phyllosilicate structure can be considered through the synthesis of phyllosilicate. Indeed, we may consider using lamellar clay compounds for radionuclide adsorption and/or structural entrapment in liquid media during or after the synthesis of these lamellar compounds.

To complete the state of art on strontium and cesium adsorption by lamellar materials, it is interesting to focus on the usage of layered double hydroxides (LDHs) for radionuclide adsorption.

### 3.2. Removal of Cs and Sr by Layered Double Hydroxides

Concerning cesium adsorption in wastewater, a study was carried out by Pshinko et al. (2015) in order to develop a LDH containing Al and Zn, sandwiched with hexacyanoferrate(II) (LDH-FeCN) [114]. This material shows excellent cesium selectivity and removes 99.8% of this radionuclide from wastewater.

Kameda et al. (2015) developed a functionalized LDH to adsorb Sr^2+^. The elaboration of a Li-Al LDH “intercalated with triethylenetetramine-hexaacetic acid (TTHA) allows this adsorption through metal chelating functions of the TTHA” [115]. Nevertheless, this LDH reveals a preferential adsorption of Nd^3+^ (with a best adsorption capability of 0.6 mmol·g^−1^) compared to Sr^2+^ (with a best adsorption capability of 0.5 mmol·g^−1^). The same team was also interested in the sorption of Sr^2+^ through Li-AL LDH interleaved with ethylenediaminetetraacetate (EDTA) and via the metal-chelating functions of this intercalated species [116]. However, this LDH has lower Sr^2+^ adsorption capacities than the Li-AL LDH sandwiched with TTHA due to the instability of the Sr-EDTA complex. Nevertheless, EDTA Li-Al LDH reveals a Nd^3+^/Sr^2+^ adsorption selectivity greater than that of TTHA Li-AL LDH.

**Figure 9:**
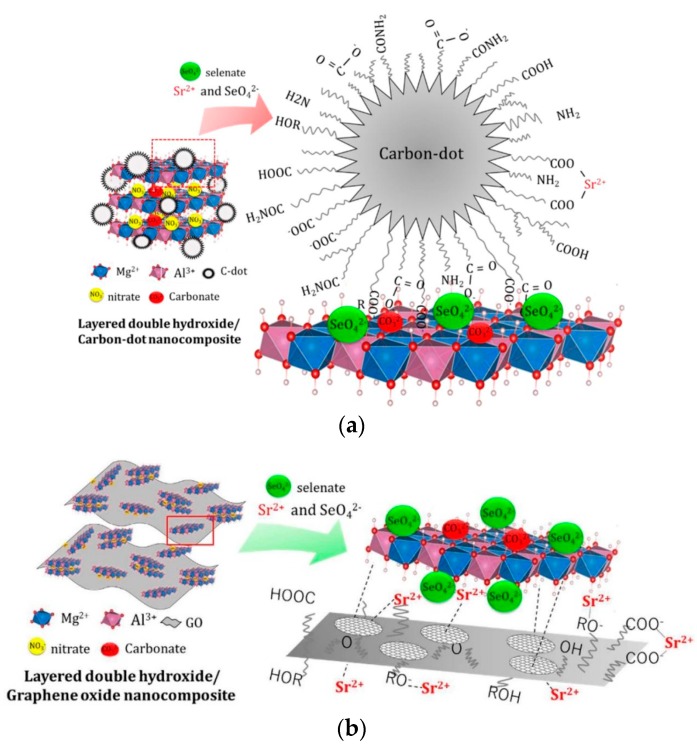
MgAl-NO_3_ LDH synthesized by Koilraj et al. and containing: (**a**) carbon nanodots [117]; and (**b**) graphene oxide [118]. Reprinted with permission from references [117,118].

Finally, a modified MgAl-NO_3_ LDH using carbon nanodots for Sr^2+^ adsorption was prepared [117], enabling a boost in the adsorption capability of the material as the quantity of carbon nanodots is increased (Figure 9a). This phenomenon is explained by a coordination between the –COO^−^ groups of carbon nanodots with Sr^2+^. The co-adsorption of SeO_4_^2−^ and Sr^2+^ on this LDH but modified with graphene oxide was also studied [118]. In this case, the Sr^2+^ adsorption capacity of this compound is 1793 mmol·g^−1^. The immobilization of Sr^2+^ is due to ionic interactions and ligand exchange with the alkoxide anion (Figure 9b).

Several LDH materials have been detailed up to now; nonetheless, the number of compositions, anions in the interlayer space and metals combinations located in the layers is nearly boundless. Thus, some researchers have been interested in the integration of radionuclides into the structure of LDH.

Nevertheless, in his review, Bravo-Suarez (2004) highlighted that, due to large differences in the ionic radii of many M^+^ metals with that of Mg^2+^ (i.e., Cs^+^, K^+^, Fr^+^, Rb^+^, Au^+^, Ag^+^ and Tl^+^ among others) [119], these M^+^ metals cannot be introduced into the LDH layers. The same issue is detected with divalent metals such as Sr^2+^ and Ba^2+^.

Indeed, only one study deals with the synthesis of Sr(II)Fe(III) “layered” double hydroxide [120]. In his thesis, Sranko tried to synthesize this material with NaOH (over 10 M) by co-precipitation method. Precursors used are strontium perchlorate trihydrate and hydrated iron perchlorate (molar Sr(II):Fe(III) ratio of the salts used is 3:1), perchloric acid used as an auxiliary agent and sodium hydroxide. The resulting material, composed of white platelets, presents a Sr(II):Fe(III) ratio of 2.91. However, even under hyperalkaline conditions, X-ray diffractograms of the solid indicated that LDHs did not appear (due to interlayer of about 6 Å that is smaller than the lower typical limit of LDHs). The author concluded that, while LDH could be formulated with strontium, they did not present a lamellar structure.

## 4. Summary, Discussions and Future Trends

Over the last 20 years, environmental requirements have become stricter, requiring high-quality of wastewater. Recently, a large variety of treatment processes such as electrodialysis, chemical precipitation, membrane filtration and adsorption have been established to remove heavy metal and radionuclide from effluent polluted by the energy industry.

Through this state of the art, we understand the great interest in using phyllosilicates as absorbing materials for toxic elements (from literature survey of more than 300 publications). Indeed, the space between clay layers is a considerable advantage since it allows the intercalation and sorption of toxic substances on it. In most investigations, adsorption studies were performed in batch process technique to determine the maximal adsorption capabilities of contaminants. To identify the clay materials effectiveness in eliminating pollutants from effluents, mechanism and adsorption rate, the parameters of isothermic adsorption, thermodynamics, kinetics, etc. are highlighted.

Several phyllosilicates highlight an excellent potential to remove toxic elements but their sorption capabilities could be enhanced by chemical modifications. In a more original way, the elaboration of new organic–inorganic hybrid compounds by sol-gel process with a phyllosilicate-like structure (mainly talc or saponite) has been considered in toxic elements adsorption. The advantages of these compounds are easiness to be prepared in water (or ethanol) in short times, they are biocompatible, their non-ecotoxicity and they have one or more (amino, thiol, etc.) functions on the carbon chain, thus improving pollutant adsorption. Synthetic phyllosilicates have usually been reported as adsorbents of copper, lead, cadmium, nickel, zinc, chromium, etc. and little information is reported on the sorption of mercury, manganese, cesium and strontium.

Table 5 outlines the best heavy metal and radionuclide adsorption capacities of these lamellar materials. These results reveal very high adsorption capacities or similar to other effective adsorbents such as carbon nanotubes [121], MoS_2_ nanosheets [122], zeolites [123], etc. Nevertheless, phyllosilicates and LDHs reveal certain limitations in terms of profitability and efficiency in the long term. Indeed, these syntheses require precursors that can be expensive and the materials obtained can be unstable with regard to certain parameters such as temperature, pressure, acidity, etc. This point is really problematic by causing potential release of pollutants. The risk assessment of these lamellar materials must also be investigated and their impact on the environment must be explored in a broader context. In the case of radionuclide adsorption, concentration of the radionuclides (coming from liquid effluents) in lamellar materials may make them emitters. It is therefore necessary to study the release of such materials by studying their behavior under radiolysis and their interactions with the storage environment.

In addition, the selectivity of the adsorbent is a parameter that is not given much prominence in these studies. It is expected that the adsorption of a noxious element may be hindered by the adsorption of other elements present in the solution. The study of the efficiency of these materials in multi-pollutant and highly diluted wastewater is strongly recommended to go further in the research towards concrete applications. In that respect, the current challenge in wastewater decontamination is to develop adsorbents that meet these four criteria: capacity, selectivity, stability and kinetics.

Water treatment by phyllosilicate or layered double hydroxide materials is currently at the laboratory scale, and research performed at small scale is necessary to evaluate the pilot scale cost effective, process development, etc.

Moreover, to date, one point has not been addressed in scientific research: radionuclides (such as cesium or strontium) integration into lamellar compounds structure. This original method would thus make it possible polluting elements to get encapsulated, trapped or complexed in the interlayer space of lamellar materials or directly integrated into their structure, making it impossible for these elements to be removed.

## Figures and Tables

**Table 1:** Maximal adsorptive efficiency of different phyllosilicates towards a variety of metal cations [3].

Heavy Metal	Adsorbent	Maximum Adsorption Capacity (mmol·g^−1^ )	Ref.
Cd (II)	Smectite	8.64	[34]
Co (II)	Chemically treated bentonite	2.34	[35]
Cr (III/VI)	Polyaniline/Montmorillonite composite	5.94	[36]
Cu (II)	Bentonite	0.85	[37]
Hg (II)	Montmorillonite	1.92	[38]
Mn (II)	Kaolinite	2.72	[39]
Ni (II)	Kaolinite	2.40	[40]
Pb (II)	Illite	1.15	[41]
Zn (II)	Kaolinite	3.82	[42]

**Table 2:** List of synthetic organic–inorganic hybrids having a phyllosilicate-like structure investigated for their adsorption characteristics with various heavy metals.

Authors	Date	Phyllosilicate Structure	Organic Compounds	Cations/Anions Adsorbed	Capacity of Cations Sorption (mmol·g^−1^)	Ref.
Fonseca et al.	2000	Talc	Thiol group	Co Ni Cu Zn	Cu^2+^ = 4.31 Ni^2+^ = 3.44 Co^2+^ =3.03 Zn^2+^ = 2.60	[54]
Fonseca et al.	2000	Mg-Talc Cu-Talc	Thiol group	Cu	Cu(Mg-Talc-Thiol) = 5.93 > Cu(Cu-talc-Thiol)	[46]
Fonseca et al.	2000	Talc	3-aminopropylamine group = SILMg1 N-propylethylenediamine group = SILMg2	Co Ni Cu Zn	Cu^2+^ [8.09, 4.53] > Zn^2+^ [6.49, 2.41] > Ni^2+^ [5.55, 3.39] > Co^2+^ [2.06, 1.24]	[61]
Fonseca et al.	2000	Ni-Talc Ni located in octahedral sites Or Ni complexed with amino groups and located in the interlayer space	Amino group	Ni	Octahedral Ni [1.78–2.65] Interlamellar Ni [1.58–2.49]	[56]
Lagadic et al.	2000	Talc	Thiol group	Hg Pb Cd	Hg = 3.01 Cd = 1.87 Pb = 1.76	[58]
Jaber et al.	2005	Saponite	Thiol group	Hg Cu Pb	Hg^2+^:100% Cd^2+^:90% Pb^2+^:50%	[59]
Sales et al.	2006	Talc	(1) Cl-group (2) 5-amino-1.3.4-thiadiazole-2-thiol molecule	Hg	Talc-2 Hg = 0.19	[44]
Moscofian et al.	2008	Talc	Nitrogen and sulfur basic centers	Cu Pb Cd	Cu = 3.28 Pb = 1.42 Cd = 0.35	[62]
Dey et al.	2009	Talc	Organosilane based on 3-glycidoxypropryltrimethoxysilane and thiourea	Cr Mn Zn	Cr(III) > Mn(II) > Zn(II)	[57]
Melo et al.	2010	Cobalt-Talc	Nitrogen and oxygen basic centers (Ethanolamines Diethanolamines)	Cu Pb	Ethanolamines: Cu = 2.01 Pb = 2.59 Diethanolamines: Cu = 2.55 Pb = 2.43	[63]
Badshah et al.	2011	Talc	Nitrogen and sulfur basic centers	Cu Pb Cd	Cu = 4.01 Pb = 7.08 Cd = 1.86	[60]
Lee et al.	2011	Talc	Amino group	CrO_4_^2−^ Fe(CN)_6_^3−^	Removal efficiency: Chromate: 89.54% Ferricyanide: 97.43%	[64]

**Table 3:** Maximum adsorptive performance (comparatively) of different LDHs towards a variety of metal cations [81].

Heavy Metal	Adsorbent (LDH)	Maximum Adsorption Capacity (mmol·g^−1^)	Ref
Cd (II)	Graphite oxide aerogels/MgAl	0.85	[88]
Cr (III/VI)	Fe^2+^/MgAl	12.5	[89]
Cu (II)	MoS_4_^2−^/MgAl	2.85	[85]
Co (II)	Polysulfide/MgAl	1.41	[86]
Hg (II)	Polysulfide/MgAl	4.05	[86]
Ni (II)	Polysulfide/MgAl	1.81	[86]
Pb (II)	CaFe_2_O_4_/polyophenylenediamine/MgAl	4.83	[90]
Zn (II)	Polysulfide/MgAl	2.22	[86]

**Table 4:** Summary of different approaches to entrap strontium on phyllosilicates, model used, type of interaction and retention capacities.

Author	Support	Model Used	Interaction Nature	Capacity
Paulus et al. [103] (1992)	Synthetic highly charged Na fluorophlogopite mica (Na_4_Mg_6_Al_4_S_4_O_20_F_4_)	Distribution coefficient K_d_ (ratio of the amount of strontium sorbed per gram of solid)	Sr^2+^ trapped into the interlayer space of the mica	2.31 mmol·g^−1^ (~44% of the theoretical exchange capacity of Na-4-mica)
Khan et al. [109] (1995)	Bentonite (from Shina Bagh. Kala Chita Forest. Attock. Pakistan)	Freundlich and Langmuir isotherms. Dubinin–Radush–Kevich (D-R) equation	Mean free energy for adsorption. E ~ 9 kJ/mol → ion exchange	sorption increases with pH (99% at pH 8.5)
Kodama et al. [104] (2001)	Synthetic Na-4-Mica	First-Order Kinetic Model Freundlich modelParabolic Diffusion Model Elovich Model	ion exchange of 2 Na^+^ by 1 Sr^2+^	0.011 mmol·g^−1^
Lu et al. [110] (2001)	Ca-montmorillonite (MMT) water collected in 1998 at DP Canyon water collected in 1994 at Fortymile Wash. (Nevada) ^85^Sr solution	Distribution coefficient K_d_	Surface complexation and isotope exchange with Ca^2+^	92–100% of the ^85^Sr quickly adsorbed
Bellenger et al. [111] (2008)	Kaolinite Montmorillonite Illite Al oxide-coating Fe oxide-coating Organic coating	Distribution coefficient K_d_		Sr adsorption similar between three phyllosilicates Kd ~ 1000 dm^3^·kg^−1^ Sr ads. ↑ by Al-coating Not affected by other coatings Adsorption irreversible
Missana et al. [105] (2008)	Illite/smectite mixtures	Ionic exchange and surface complexation modeling (non-electrostatic model)	Single clay: Ionic exchange	pH [2,3,4,5,6,7,8,9] Kd = 4580 mmol·g^−1^ pH > 9 Kd = 4000–11500 mmol·g^−1^
Galambos et al. [112] (2009)	Slovak bentonites	Langmuir isotherm	Cation-exchange mechanism	Sr sorption ↑ when: pH ↑ metal C°↓
Galambos et al. [113] (2012)	Slovak bentonites: Montmorillonite Saponite Hectorite	Distribution coefficient Adsorption percentage Adsorption capacity	Basal surface and edges sites: Cation-exchange mechanism	The authors advised against using Fe-rich smectite to store radioactive waste
Wu et al. [108] (2012)	Organo-montmorillonites: Ca-Mt APTES-Mt: grafted with 3-aminopropyl triethoxysilane SDS-Mt: surface modified by sodium dodecyl sulfate HDTMAB-Mt: surface modified by hexadecyl trimethyl ammonium bromide	Pseudo-second-order model SDS-Mt: DKR and Langmuir models Ca-Mt and APTES-Mt: Freundlich model for	Ca-Mt: ion exchange SDS-Mt: surface adsorption APTES-Mt: ligand adsorption	APTES-Mt: 0.75 mmol·g^−1^ Ca-Mt:0.15 mmol·g^−1^ SDS-Mt:0.3064 mmol·g^−1^ HDTMAB-Mt: 0.04 mmol·g^−1^
Yu et al. [106] (2015)	Na-Montmorillonite	Diffuse-layer model (Sr sorption simulation) Langmuir and Freundlich models (sorption isotherms simulations)	At low pH: outer-sphere surface complexation and ion exchange. At high pH: inner-sphere surface complexation	Sorption capacity of 0.12 mmol·g^−1^
Siroux et al. [107] (2017)	MX80 bentonite (purified and conditioned under Na-saturated)	Multi-site ion exchange model. Ion exchange theory	2Na^+^/Sr^2+^ exchange	0.886 mmol·g^−1^

**Table 5:** Summary of best adsorption capacities of heavy metals and radionuclides by phyllosilicates and LDHs.

Noxious Element	Adsorbent	Maximum Adsorption Capacity (mmol·g^−1^)	Ref
**Heavy Metals**			
Cd (II)	Smectite	8.64	[34]
Cr (III/VI)	Fe^2+^/MgAl (LDH)	12.5	[89]
Cu (II)	Synthetic Talc with Nitrogen basic centers	8.09	[61]
Co (II)	Synthetic Talc with Thiol group	3.03	[54]
Hg (II)	Polysulfide/MgAl (LDH)	4.05	[86]
Ni (II)	Kaolinite	2.4	[40]
Pb (II)	Synthetic Talc with Nitrogen and sulfur basic centers	7.08	[60]
Zn (II)	Synthetic Talc with Nitrogen basic centers	6.49	[61]
**Radionuclides**			
Cs	Montmorillonite	0.78	[114]
	Vermiculite	0.27	
	Illite	0.15	
Sr	Synthetic Na-4-Mica	2.31	[103]
	MgAl-NO_3_ (LDH)	1.79	[118]
